# Phylogeography and larval spine length of the dragonfly *Leucorhinia dubia* in Europe

**DOI:** 10.1371/journal.pone.0184596

**Published:** 2017-09-13

**Authors:** Frank Johansson, Peter Halvarsson, Dirk J. Mikolajewski, Jacob Höglund

**Affiliations:** 1 Department of Ecology and Genetics, Uppsala University, Uppsala, Sweden; 2 Institut für Biologie, Freie Universität Berlin, Berlin, Germany; National Cheng Kung University, TAIWAN

## Abstract

Presence or absence of predators selects for different kind of morphologies. Hence, we expect variation in traits that protect against predators to vary over geographical areas where predators vary in past and present abundance. Abdominal larval spines in dragonfly larvae provide protection against fish predators. We studied geographical variation in larval spine length of the dragonfly *Leucorrhinia dubia* across Western Europe using a phylogenetic approach. Larvae were raised in a common garden laboratory experiment in the absence of fish predators. Results show that larvae from northern Europe (Sweden and Finland) had significantly longer larval spines compared to larvae from western and central Europe. A phylogeny based on SNP data suggests that short larval spines is the ancestral stage in the localities sampled in this study, and that long spines have evolved in the Fenno-Scandian clade. The role of predators in shaping the morphological differences among the sampled localities is discussed.

## Introduction

Spatial environmental variation act as a major source of divergent natural selection resulting in phenotypic diversification within species and given enough time, might result in speciation [1]. Predators represent a strong selective force, with abundance and type of predators showing strong variation across environments [2]. Prey species have evolved a richness of highly effective morphological anti-predators traits to avoid and repel predators [3, 4]. Because many morphological anti-predator traits are costly to produce, express and maintain, net differences between antipredator trait costs and benefits only turn positive in the presence of predators [5]. Thus, differences in predator abundance and occurrence are predicted to result in variation in anti-predator morphological expression among and within prey species [6]. Such variation could occur at larger geographical scales [7], and a good understanding of the geographical variation in morphological traits that defends against predators will contribute to a thorough understanding on how trait diversification evolves [6].

Phylogeography, the study of geographic distribution of genetic lineages [8], is a useful approach to understand processes that have resulted into phenotypic differentiation amongst populations. By mapping trait differences between populations on a phylogeny, an understanding of the rate and variation of such diversification can be achieved. For example, by mapping body morphology of *Anolis* lizards on a phylogeny, Losos et al. [9] showed that morphological diversification stemmed from several independent events across islands resulting into convergent phenotypes. Less well studied is the diversification of anti-predator traits across lineages, but see e.g. Mikolajewski et al. [10] and Ge et al. [11]. Among anti-predator defenses, morphological defenses such as spines and spikes have been at the center of interest for decades. They represent prominent features to repel predators when already detected and attacked by a predator, and such traits are present both in animals and plants [12, 13, 14, 15]. In this study, we focus on the constituent part of the phenotypic variation in larval spine length across Europe in larvae of a dragonfly (Odonata) species. Odonate larvae express prominent abdominal spines that feature as anti-predator traits, with selection by predatory fish shaping occurrences and length of spines. The variation in spine morphology represent an ideal system to understand how selection shapes anti-predator traits [16], however, how patterns of variation in spines occurrence evolved at the intra-specific level is less well understood.

Larvae of the dragonfly *Leucorrhinia dubia (Vander Linden*, *1825)* express abdominal spines, but show large intra as well as inter-population variation in dorsal and lateral abdominal spine length [17, 18]. Abdominal spines reduce predation risk, because fish have a longer handling time when eating long-spined larvae [17]. In addition, studies on other *Leucorrhinia*-species have shown that larvae with longer spines have higher rejection rates after an attack [14, 15]. Parts of the inter-population variation in spines length among individuals stems from longer spines being phenotypically induced by the presence of predatory fish [18, 19]. However, there is also strong non-plastic variation in spine length among individuals within and among lakes with and without fish. For example, some none-fish lakes have larvae with a mean spine length larger than that of some fish lakes [16], and this spine length variation occurs at a micro-geographical scale of a radius of about 100 km. However, at a larger geographical scale we do not know how intraspecific larval spine length varies and how this variation has evolved. One way to study how spines as a defensive trait have evolved is to map existing trait variation on a phylogeny. To study predator morphological defense evolution within *L*. *dubia* we first raised larvae from six sampled locations across Europe from the egg stage in the absence of fish under laboratory conditions. This gave us a good estimate of the present variation in larval spine length across *L*. *dubi*a’s main distribution in Europe. Second, we reconstructed a phylogeny based on independent SNP-data, using individuals sampled from 9 locations across Europe and mapped larval spines length on the corresponding tree. By this, we detected variation in spine length on which natural selection via predation can work as well as reveal how spines arise as an effective defense against predatory fish.

## Methods

### Ethics statement

*L*. *dubia* has a wide distribution and is common in the northern parts of its range in Europe. It is categorized as least concern in Europe by International Union for Conservation of Nature (IUCN), and the species is abundant at all the localities sampled in this study. Permits for sampling Odonata at the localities in United Kingdom and Belgium was by provided by Natural England and ANB-Flanders (Agentschap voor Natuur en Bos) respectively.

### Spine length variation among populations across Europe

To examine spine length among European populations of *L*. *dubia* we reared larvae in a common garden experiment in the laboratory. Eggs from 5 females from 6 localities without fish, across Europe (United Kingdom, Germany, Austria, Poland, Sweden, and Finland) were collected by dipping the females’ abdomen into glass jars filled with water from the lake/pond of collection. Fish status was determined by several factors. First, repeatedly netting in at the localities did not reveal any fish. Second many of the sampled ponds/lakes are small and freeze all the way to the bottom in the winter which kills all fish. Third, frequent communication with local fishermen confirmed the absence of fish in the localities sampled. Eggs clutches were thereafter brought to the laboratory where they were kept in 1.0 l. plastic containers filled with non-chlorinated tap water. Eggs from all females hatched after 2–3 weeks. Upon hatching we mixed larvae from all females from one locality into a larger container and then randomly picked 25 larvae from this pool of larvae for the experiment. The experiment was run in 0.5 l containers filled with 0.3 l non-chlorinated tap water. Larvae were raised individually in these containers and fed daily *ad libitum* with a mixture of *Artemia* nauplii and daphnids from laboratory cultures. The temperature was 20°C and the day/night cycle was 14 hour light/10 hour dark.

The rearing ended when larvae had reached their final instar. At this stage they were preserved in 80% alcohol for subsequent measurements of larval size and spine length. These measurements were taken using a dissecting microscope with an ocular micrometer by placing the larvae in a petri dish with alcohol. Size was estimated as head width, representing the outermost points on each eye when the larva was viewed from above. This length is a reliable measure of size in dragonfly larvae [20]. Length of dorsal spine number 4–7 and lateral spine 8–9 was taken as in Johansson [19].

Because spine lengths show patterns of multicollinearity within individuals, we performed a principal component analyses (PCA) on the covariance matrix using all spine length measures to reduce number of variables. The first PC axis explained the majority of the variation (PC1 = 0.75, PC2 = 0.12, PC3 = 0.07) and therefore we retained the scores from this PC-axis and used them in subsequent analyses as the spine length of each individual. We corrected spine length for larval size by dividing spine length by larval size. Then we run an ANOVA on spine length using all six sampling location as a factor, followed by a Turkey’s test for pairwise comparisons. Residual error distribution of spine length did not deviate from normality. In addition, we run an ANCOVA with sampling location as factor, larval size as covariate and PC1 scores un-corrected by larval size as response variable.

### Phylogeny

To examine the phylogenetic relationship among the six populations used for spine length estimates we sampled adults and larvae from these locations in addition to three more locations, resulting in a total of nine locations sampled (n range = 5–13 individuals, Table 1). Note that we did not raise larvae for spine length estimates from the additional localities: France, Belgium and Switzerland.

**Table 1:** Name and coordinates of locations sampled, n denotes number of individuals sampled. The sample from Poland is a pooled sample from four localities treated as one population (54°02'45'' N 17°52'45''E, 53°54.37 N, 16°41.65'E, 54°02'18 N, 17°51'03 E, 54°23'14'' N 17°58'00''E).

Population	Coordinates	n
United Kingdom, Chartley Moss NNK	52° 51' 04''N, 01° 58' 06''E	13
France, Stany del Recó Pyrenees	42° 33' 14''N, 02° 00' 30"E	5
Belgium, Naturo Reserv de Maten N	50° 57' 02''N, 05° 26' 58"E	9
Germany, Blankesmoor, Leifered	52° 28' 10''N, 10° 25' 26"E	11
Switzerland, Paluds dels Pelets	46° 29' 25''N, 10° 28' 34''E	5
Austria, Schwartzer See	46° 52' 25''N, 10° 28' 34''E	5
Poland, Loryniec and Koscierzyna	54° 02' 45''N, 17° 52' 45''E	10
Sweden, Grössjön	63° 47' 29"N, 20° 22' 01"E	7
Finland, Pieni Luotojärvi	67° 07' 03"N, 24° 52' 33"E	13

The samples were stored in 80% ethanol after collection awaiting DNA extraction. DNA was extracted using a modified high salt protocol [21], from either flight muscle in adults or abdominal muscles of last instar larvae. Double Digest Restriction Associated DNA (ddRAD) library data was created using a modified version of protocols from Parchman et al. [22], Peterson et al. [23] and Mastretta-Yanes et al. [24]. In short, DNA was cut by the enzymes EcoRI-HF and MseI, individual tags and primer sites ligated with T4 DNA ligase and PCR was conducted with Q5 DNA polymerase (New England Biolabs, Massachusetts, USA), see Mastretta-Yanes et al. [24] for full details. To avoid PCR bias, each sample was used four times in individual PCRs, and randomly located on the PCR plate. Each sample was uniquely tagged. After PCR, the samples and replicates were pooled and size selections were performed in the same gel. The library was then sequenced in a single separate lane on an Illumina HiSeq200 from both directions (2x125bp) in high throughput mode at SciLifeLab, Uppsala, Sweden. STACKS v1.34 [25] was used to quality filter and demultiplex samples. The demultiplexed samples were *de novo* assembled using STACKS and one SNPs per stack were extracted for the dataset. In total 1674 SNP was used for the subsequent analysis.

To create a phylogeny of the nine sampled populations, the data set was imported into BEAUti v2.3.2 [26], where the data were prepared for analyses with the SNAPP v. 1.2 plugin [27] in BEAST v. 2.4.1 [26]. Parameters were set at default values, e.g. coalescent rate k = 10, and h defined by a c prior with shape parameter α = 11.75 and scale parameter β = 109.73. Priors for forward (u) and reverse (v) mutation rates were set to be estimated. BEAST runs were carried out for 297,000 generations and the chain was sampled every 250 generations. Tracer v. 1.6 [28] was used to assess convergence and the run was considered acceptable when the effective sample size (ESS) value was over 200. A low ESS means that the trace contains a lot of correlated samples and does not represent the posterior distribution well. In our analysis the ESS was 456, see supportive information, S1 File. Output was visualized in Densitree v. 2.2.4 [29]. Burn-in was set to 10% of the samples. Finally, we mapped spine length visually on the phylogram. This was done by using the score value from the PCA on spine length.

## Results

### Spine length variation among populations across Europe

**Fig 1:**
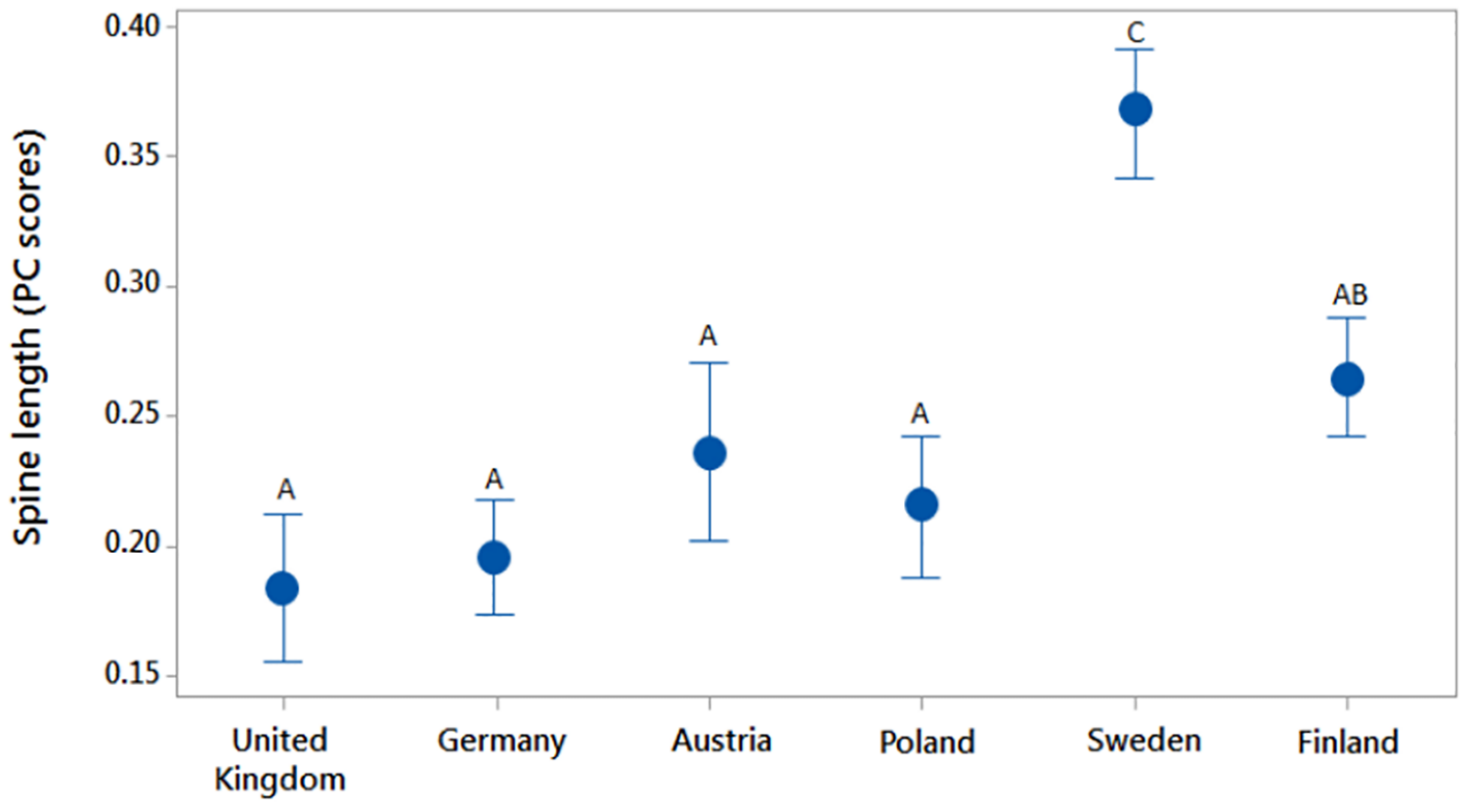
Abdominal spine length (PC scores) of larvae from six localities across Europe when raised in a common environment in the laboratory. Error bars with 95% confidence intervals. Letters above confidence interval bars denote statistically significant differences between treatments (Tukey test, p < 0.05).

Larvae took between 175 and 394 days to reach the final instar, but there was no correlation between larval spine length and duration to reach final instar (r^2^ = 0.23, d.f. = 62, P = 0.24). Spine length differed between sampled localities (F_5,67_ = 34.6, P < 0.001) (Fig 1). A subsequent Turkey’s test showed that larvae from the Swedish localities had significantly longer spines than larvae from all other localities (P < 0.001), and that larvae from the Finnish locality had significantly longer spines than those from Germany and United Kingdom (P < 0.001). All other pairwise comparisons did not differ significantly (P > 0.07). The ANCOVA using larval size as a covariate showed qualitatively similar results with an overall significant sampling site effect (F_5,63_ = 22.3, P = 0.003).

### Phylogeny

**Fig 2:**
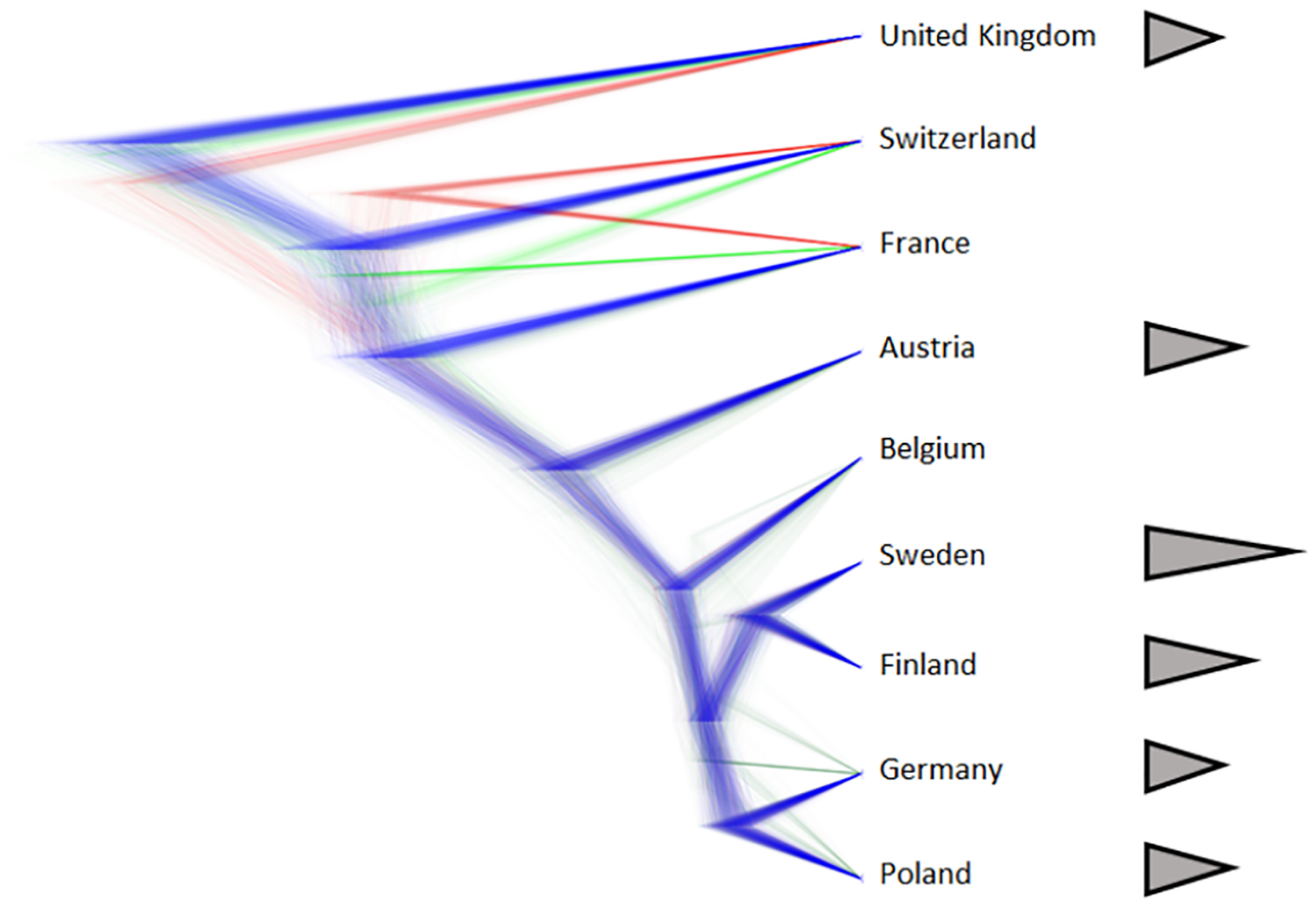
Relationships among the 9 sampled *Leucorrhinia dubia* populations across Europe based on 1674 SNPs from ddRAD sequence data. Thin lines represent densities that represent the branches. Alternative topologies are drawn in different color, where blue represent the most supportive, followed by red and green. The triangles to the right represent cartoons of larval spine length showing the proportional differences in length. Note that in reality the longest spines are also wider at the base.

The SNAPP analyses recovered a tree that revealed one well-supported clade and some basal uncertainties. The well-supported clade consisted of the sampled locations in Poland, Germany, Finland, Sweden, Belgium and Austria (Fig 2). The locations in England, Switzerland and France formed a basal grouping, with somewhat less congruence since some alternative branching pattern occurred (Fig 2). Nevertheless, the sampled English location appears to belong to a separate lineage, and the Swiss and French sampled localities seem to be less supported compared to the other lineages (Fig 2).

The Swedish and the Finnish sampled population had the longest spines and these two localities group together in the tree. These two sampled localities were however nested within the well-supported clade in which the larvae from the Germany/Poland clade had evolved shorter spines lengths (Fig 2). In summary, in our sampled localities short spines was the ancestral stage and the localities in Sweden and Finland have evolved longer spines.

## Discussion

Our study on the morphological variation in larval spine length in *L*. *dubia*, consisted of a small, but wide spread sample across Western Europe. Variation in the expression of defensive spines can stem from genetic differentiation as well as phenotypic plasticity. Since we used a common garden approach we can exclude phenotypic plasticity as a source of variation of spine length for our result on *L*. *dubia*. We found that spine length differed among populations, and with populations in Northern Europe (Sweden & Finland) having evolved considerably longer abdominal spines. Large parts of the variation in abdominal spine length is associated with the presence/absence of fish predators within and among species of *Leucorrhinia*, such that populations and species that co-occur with fish on average are having longer spines [17, 18, 30]. In addition, theses larval spines are adaptive, since they provide protection against fish predator attacks [14, 15, 17]. We therefore suggest that the difference in spine length among populations is driven by natural selection by predatory fish, and fish abundance in our study area is discussed below. Patterns of differential predation regimes that drive variation in spine length has been found in allopatric populations of sticklebacks [31, 32], and experimental evidence showed that this differentiation is due to natural selection from fish predators [33]. Direct experimental evidence that natural selection by fish predators cause selection for longer spines is however lacking for *L*. *dubia* larvae. But direct experimental evidence has shown that invertebrate predators, which are the dominant predators in fishless lakes select for shorter spines in congeneric *L*. *caudalis* larvae [34].

*Leucorrhinia dubia* and other dragonfly species disperse for quite large distances covering more than 1 km in the adult stage [35, 36], and genetic studies show little genetic differentiation in many species, e.g. Damm and Hadrys [37] and Johansson et al. [38], suggesting that dispersal between pond and lakes is common. Thus selection for longer spines that increase survival from fish predation is probably acting over a larger area than the size of a pond or lake. We suggest that *L*. *dubia* is relatively more frequently in lakes with fish in Northern European countries (Sweden and Finland) compared to the rest of the area we sampled, even though absolute abundance of *L*. *dubia* is higher in lakes without fish in the north [39]. For example *L*. *dubia* do occur in much higher densities in Northern Europe, and is commonly found in bog ponds without predatory fish and as well as lakes with predatory fish [39], but note that our northern samples came from ponds without fish. In contrast, occurrence of *L*. *dubia* in the rest of Europe seems to be restricted to small bog ponds without predatory fish [40, 41, 42]. However, quantitative data are needed to confirm this suggestion as we have no data on the abundance and diversity of fish in the sampled areas. Furthermore, we sampled only two location in the northern area where *L*. *dubia* is abundant and do occur with fish, although in lower abundance. Had we sampled more localities in these region we predict that these population should show long larval spine length as was shown by Johansson and Samuelsson [17] who sampled 7 lakes with fish in Northern Sweden. We would also predict a low population differentiation between northern populations compared to populations is central and southern Europe, since high population density [43] and dispersal would cause less differentiation in the north.

Among *Leucorrhinia*-species with defensive spines, *L*. *dubia* is categorized as a short-spined and non-fish lake species [30, 44]. The two populations from Northern Europe which had the longest larval spines were nested within the Central European populations, suggesting that long spines have evolved from shorter spine length in the ancestor of this lineage, and that the short spines is the ancestral stage. However, a sample size including more populations might reveal a different ancestral stage. A phylogeny covering all 14 currently accepted species of the genus *Leucorrhinia* showed that long larval spine represents the ancestral stage among these species [44], and the authors suggested that spines were lost as populations invaded fish free areas. Thus, the among species comparison [43] and the within species comparison we present here suggest that larval spine length can evolve in both directions (shorter and longer) within a genus during evolutionary time scales.

Apart from genetically determined spine length differences between individuals, spine length in *L*. *dubia* is also plastic such that longer spines can be induced by predatory fish cues [19]. Many studies show a genotype by environment interaction at the population level and also a population by environment interaction with regard to induced defenses, i.e. individuals and populations differ in their degree of plasticity [19, 45, 46], but see [47]. Therefore, we cannot be sure that the relative spine length differences observed among the populations would show the same patterns if larvae had been raised in the presence of fish. However, under the assumption that spine length is an adaptation we see no obvious reason for why spine length should be shorter in the presence of fish areas with high abundance of fish relative to populations from areas without fish.

In summary, our study show that spine lengths in *L*. *dubia* vary geographically across Western Europe and it suggests that longer spines have evolved from shorter spines in the studied clade. Whether the variation across the geographical scale is related to the presence of fish across the landscape awaits further investigations.

## Supporting information

S1 File Table and figures with diagnostics from Tracer. (DOCX)

## References

[1] Schluter D. The ecology of adaptive radiation. 2000 Oxford University Press.

[2] Wellborn GA, Skelly DK, Werner EE. Mechanisms creating community structure across a freshwater habitat gradient. Annual Review of Ecology and Systematics. 1996; 27: 337–363.

[3] Edmunds M. Defence in animals. 1974 Longman, New York.

[4] Tollrian R, Harvell CD. The ecology and evolution of inducible defenses. 1999 Princeton University Press, Princeton, N.J.

[5] Abrams PA. Character shifts of prey species that share predators. American Naturalistt. 2000; 156 (Suppl.): S45–S61. 10.1086/303415 29592581

[6] Vamosi SM. On the role of enemies in divergence and diversification of prey: a review and synthesis. Canadian Journal of Zoology. 2005; 894–410.

[7] Large SI, Smee DL. Biogeographic variation in behavioral and morphological responses to predation risk. Oecologia. 2013; 171: 961–9 doi: 10.1007/s00442-012-2450-5 23001623 10.1007/s00442-012-2450-5

[8] Avise JC. Phylogeography: The History and Formation of Species. Harvard University Press, Cambridge 2000 MA. 447 pp.

[9] Losos JB, Jackman TR, Larson A, Queiroz K, Rodriguez-Schettino L. Contingency and determinism in replicated adaptive radiations of island lizards. Science. 1998; 279: 2115–2118. 9516114 10.1126/science.279.5359.2115

[10] Mikolajewski DJ, De Block M, Rolff J, Johansson F, Beckerman AP, Stoks R. Predator-driven trait diversification in a dragonfly genus: covariation in behavioural and morphological antipredator defense. Evolution. 2010; 64: 3327–3335. doi: 10.1111/j.1558-5646.2010.01078.x 20624175 10.1111/j.1558-5646.2010.01078.x

[11] Ge D, Chesters D, Gomez-Zurita J, Zhang L, Yang X, Vogler AP. Anti-predator defence drives parallel morphological evolution in flea beetles. Proceedings of the Royal Society B: Biological Sciences. 2011; 278:1715, 2133–2141. 10.1098/rspb.2010.1500 PMC3107618 21159678

[12] Havel JE, Dodson SI. *Chaoborus* predation on typical and spined morphs of *Daphnia pulex*: behavioural observation. Limnology and Oceanography. 1984; 29: 487–494.

[13] Cooper SM, Owen-Smith N. Effects of plant spinescence on large mammalian herbivores. Oecologia. 1986; 68: 446–455. doi: 10.1007/BF01036753 28311793 10.1007/BF01036753

[14] Mikolajewski DJ, Rolff J. Benefits of morphological defence demonstrated by direct manipulation in larval dragonflies. Evolutionary Ecology Research. 2004; 6: 619–626.

[15] Mikolajewski DJ, Johansson F. Morphological and behavioral defenses in dragonfly larvae: trait compensation and co-specialization. Behavioral Ecology. 2004; 15: 614–620.

[16] Johansson F, Mikolajewski DJ. Evolution of morphological defences In Córdoba-Aguilar A. (ed.) Dragonflies: Model Organisms for Ecological and Evolutionary Research. 2008 Oxford University Press, pp 127–138.

[17] Johansson F, Samuelsson L. Fish induced variation in spine length of *Leucorrhinia dubia* (Odonata) larvae. Oecologia. 1994; 100: 74–79 doi: 10.1007/BF00317132 28307029 10.1007/BF00317132

[18] Johansson F. Reaction norms and costs of plasticity of a predator induced morphological defence in a larval dragonfly (*Leucorrhinia dubia*: Odonata). Canadian Journal of Zoology. 2002; 80: 944–950.

[19] Johansson F, Wahlström E. Induced morphological defence: evidence from whole lake manipulation experiments. Canadian Journal of Zoology. 2002; 80: 199–206.

[20] Benke AC. A method for comparing individual growth rates of aquatic insects with special reference to Odonata. Ecology. 1970; 51: 328–331.

[21] Paxton RJ, Thoren PA, Tengö J, Estoup A, Pamilo P. Mating structure and nestmate relatedness in a communal bee, *Andrena jacobi* (Hymenoptera, Andrenidae), using microsatellites. Molecular Ecology. 1996; 5: 511–519. 8794560 10.1111/j.1365-294x.1996.tb00343.x

[22] Parchman TL, Gompert Z, Mudge J, Schilkey FD, Benkman CW, Buerkle CA. Genome-wide association genetics of an adaptive trait in lodgepole pine. Molecular Ecology. 2012; 21: 2991–3005. doi: 10.1111/j.1365-294X.2012.05513.x 22404645 10.1111/j.1365-294X.2012.05513.x

[23] Peterson BK, Weber JN, Kay EH, Fisher HS, Hoekstra HE. Double Digest RADseq: An Inexpensive Method for De Novo SNP Discovery and Genotyping in Model and Non-Model Species. PLoS ONE. 2012; 7(5), e37135 doi: 10.1371/journal.pone.0037135 22675423 10.1371/journal.pone.0037135 PMC3365034

[24] Mastretta-Yanes A, Arrigo N, Alvarez N, Jorgensen TH, Piñero D, Emerson B C. Restriction site-associated DNA sequencing, genotyping error estimation and de novo assembly optimization for population genetic inference. Molecular Ecology Resources. 2015; 15: 28–41. doi: 10.1111/1755-0998.12291 24916682 10.1111/1755-0998.12291

[25] Catchen J, Hohenlohe PA, Bassham S, Amores A, Cresko WA. Stacks: An Analysis Tool Set for Population Genomics. Molecular Ecology. 2013; 22: 3124–40. doi: 10.1111/mec.12354 23701397 10.1111/mec.12354 PMC3936987

[26] Bouckaert R. & Heled J. DensiTree 2: Seeing Trees Through the Forest bioRxiv. 2014 http://dx.doi.org/10.1101/012401

[27] Bryant D, Bouckaert R, Felsenstein J, Rosenberg NA, RoyChoudhury A. Inferring species trees directly from biallelic genetic markers: bypassing gene trees in a full coalescent analysis. Molecular Biology and Evolution. 2012;29:1917–1932. doi: 10.1093/molbev/mss086 22422763 10.1093/molbev/mss086 PMC3408069

[28] Rambaut A, Suchard MA, Xie D, Drummond AJ. Tracer v1.6. 2014 http://beast.bio.ed.ac.uk/Tracer

[29] Bouckaert R, Heled J, Kühnert D, Vaughan T, Wu C-H, Xie D et al BEAST 2: A Software Platform for Bayesian Evolutionary Analysis. PLoS Computational Biology. 2014; 10(4), e1003537 doi: 10.1371/journal.pcbi.1003537 24722319 10.1371/journal.pcbi.1003537 PMC3985171

[30] Petrin Z, Schilling E, Loftin C, Johansson F. Predators shape distribution and promote diversification of morphological defenses in Leucorrhinia, Odonata. Evolutionary Ecology. 2010; 24:1003–1016

[31] Walker JA. Ecological morphology of lacustrine threespine stickleback *Gasterosteus aculeatus* L. (Gasterosteidae) body shape. Biological Journal of the Linnean Society. 1997; 61: 3–50.

[32] Walker JA, Bell MA. Net evolutionary trajectories of body shape evolution within a microgeographic radiation of threespine sticklebacks (*Gasterosteus aculeatus*). Journal of Zoology (Lond.). 2000; 252: 293–302.

[33] Vamosi SM, Schluter D. Impacts of trout predation on fitness of sympatric sticklebacks and their hybrids. Proceedings of the Royal Society B: Biological Sciences. 2002; 269: 923–930 doi: 10.1098/rspb.2001.1946 12028775 10.1098/rspb.2001.1946 PMC1690976

[34] Mikolajewski DJ, Johansson F, Wohlfart B, Stoks R. Invertebrate selection selects for the loss of a morphological antipredator trait. Evolution. 2006; 60: 1306–1310. 16892980

[35] Pajunen VI. Studies on the population ecology of *Leucorrhinia dubia* V. d. Lind. (Odon., Libellulidae). Annales Zoologici Societatis Zoologicae-botanicae Fennicae 'Vanamo'. 1962; 24: 1–79.

[36] McCauley SJ. The role of local and regional processes in structuring larval dragonfly distributions across habitat gradients. Oikos. 2007; 116: 121–133.

[37] Damm S, Hadrys H. A dragonfly in the desert: genetic pathways of the widespread *Trithemis arteriosa* (Odonata: Libellulidae) suggest male-biased dispersal. Organism Diversity and Evolution 2012; 12: 267–279.

[38] Johansson F, Halvarsson P, Mikolajewski DJ, Höglund, J. Genetic differentiation in the boreal dragonfly Leucorrhinia dubia in the Palearctic region. Biological Journal of the Linnean Society. 2017; 121 294–304.

[39] Johansson F, Brodin T. Effects of fish predators and abiotic factors on dragonfly community structure. Journal of Freshwater Ecology. 2003; 18: 415–423.

[40] Kognitz S. Kleine Moosjungfer In: Libellen in Bayern. (eds. Kuhn K. & Burbach K.). 1998 Verlag Eugen Ulmer, Stuttgart.

[41] Wildermuth H, Gonseth Y, Maibach A. Odonata—Die Libellen der Schweiz, Fauna Helvetica 12, CSCF/SEG, 2005. Neuchâtel.

[42] Wittwer T, Sahlén G, Suhling F. Does one community shape the other? Dragonflies and fish in Swedish lakes. Insect Conservation and Diversity. 2010; 3: 124–133.

[43] Boudot J-T, Kalkman VJ. Atlas of the European dragonflies and damselflies. 2015 KNNV Publishing, the Netherlands.

[44] Hovmöller R, Johansson F. A phylogenetic perspective on larval spine morphology in *Leucorrhinia* (Odonata: Libellulidae) based on ITS1, 5.8S and ITS2 rDNA sequences. Molecular Phylogenetics and Evolution. 2004; 30: 653–662. doi: 10.1016/S1055-7903(03)00226-4 15012945 10.1016/S1055-7903(03)00226-4

[45] Trussell GC, Smith LD. Induced defenses in response to an invading crab predator: an explanation of historical and geographic phenotypic change. Proceedings of the National Academy of Science. USA. 2000; 97: 2123–2127. 10.1073/pnas.040423397 PMC15764 10681425

[46] Kishida O, Trussell GC, Nishimura K. Geographic variation in a predator‐induced defense and its genetic basis. Ecology.2007; 88: 1948–1954. 17824425 10.1890/07-0132.1

[47] Brönmark C, Lakowitz T, Hollander J. Predator-induced morphological plasticity across local populations of a freshwater snail. PLoS One. 2011; 6(7): e21773 doi: 10.1371/journal.pone.0021773 21818264 10.1371/journal.pone.0021773 PMC3139574

