# Supplementary material for: Phylogeography and larval spine length of the dragonfly Leucorhinia dubia in Europe
Source: PLoS One. 2017 Sep 13;12(9):e0184596. doi: 10.1371/journal.pone.0184596 (PMC5597221; doi:10.1371/journal.pone.0184596)
Supplement: S1 File — (DOCX) [file pone.0184596.s001.docx]

**S1 File**

The following table and figures contain diagnostics from Tracer^1^. The SNAPP run length is 297000 states, 27900 burn-in.

^1^ Rambaut A, Suchard MA, Xie D & Drummond AJ (2014) Tracer v1.6, Available from <http://beast.bio.ed.ac.uk/Tracer>

**Table S1.** Summary statistics for the SNAPP analysis in BEAST. The table shows descriptive statistics including mean, stdev, 95% confidence intervals and other statistics for the analysis.

| Summary Statistic | Posterior distribution |
| --- | --- |
| mean | -52541.9011 |
| stderr of mean | 0.1078 |
| stdev | 2.3025 |
| variance | 5.3017 |
| median | -52541.4894 |
| 95% HPD Interval | [-52546.1486, -52537.6257] |
| auto-correlation time (ACT) | 586.9622 |
| effective sample size (ESS) | 456.1622 |

Auto-Correlation Time (ACT) = Number of states, in the MCMC chain, that two samples have to be from each other for them to be uncorrelated. ACT is estimated from the samples in the trace excluding burn-in.

Effective sample Size (ESS) = Number of independent samples the trace is equivalent to.


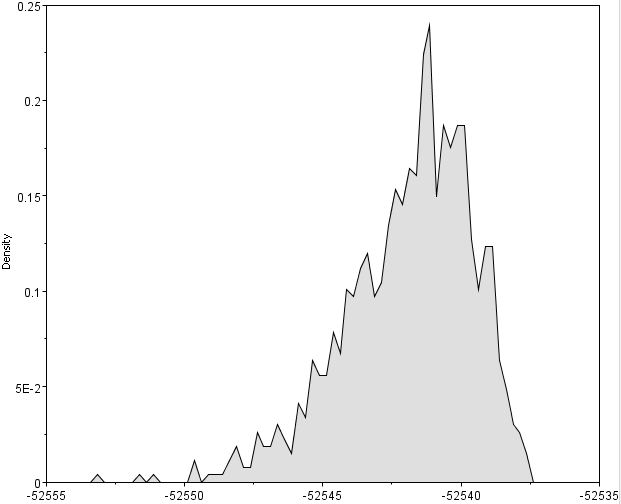


**Figure S1.** Posterior probability density has a bell-shaped curve. There’s a good estimate of the mean and credible intervals (see Table S1), however longer chain length could reduce the stochastic noise.


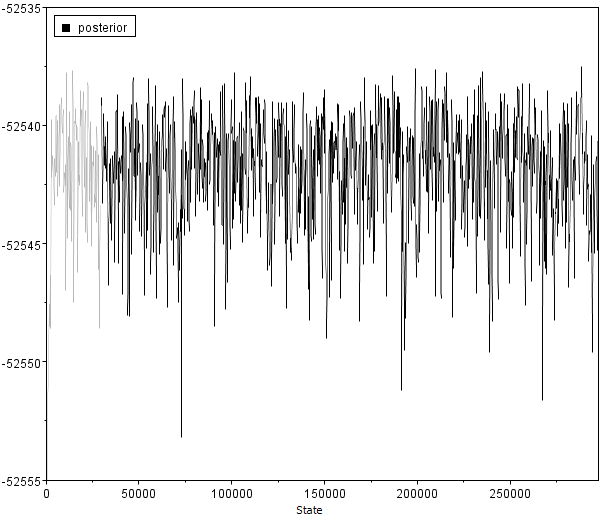


**Figure S2.** Raw trace plot. There are no obvious trends in the plot, suggesting that the MCMC has converged. Also, there are no large scale fluctuations in the trace that could suggest poor mixing. Gray traces are burn-in and black traces are sampled traces.
